# A New Method for Plastic Closure of an Extensive Laryngotracheal Defect

**DOI:** 10.22038/IJORL.2022.60491.3084

**Published:** 2022-05

**Authors:** Liliya V. Selezneva, Svetlana V. Starostina, Alexey V. Toldanov, Emil N. Sobol, Olga I. Baum, Valeriy M. Svistushkin

**Affiliations:** 1 *Depaerment of Ear, Nose and Throat Diseases, I.M. Sechenov First Moscow State Medical University, Moscow, Russia.*; 2 *Institute of Photonic Technologies, Federal Scientific Research Centre ‘Crystallography and Photonics’of Russian Academy of Sciences, Troitsk, Moscow 142190, Russia.*

**Keywords:** laryngeal stenosis, laryngotracheal defects, laser modeling of cartilage tissue

## Abstract

**Introduction:**

Elimination of extensive defects of the larynx and trachea by using musculoskeletal plastics without the use of supporting materials is not always sufficient. Laser modeling of cartilage tissue is a promising technique in modern medicine.

**Case Report:**

This article presents a new method for plastic closure of an extensive defect in the larynx and trachea with the help of costal auto-cartilage modeled by an erbium fiber laser with wavelength of 1.56 μm.

**Conclusions:**

The presented method allows us to restore the anatomical integrity of the respiratory tract at the final stage of surgical treatment of patients with chronic combined laryngeal and tracheal stenosis. Presented own clinical observation.

## Introduction

The increase in the number of patients with chronic-combined stenosis of the larynx and trachea is primarily due to the increase in trauma to recurrent laryngeal nerves during complex surgeries on the organs of the head and neck, long-term artificial ventilation of the lungs, traffic accidents with numerous severe craniocerebral and neck injuries (1,2). Nowadays development of new methods of reconstructive interventions for paralytic and combined stenosis of the larynx and trachea remains an urgent task. 

The main goal of open reconstructive and restorative surgeries on the larynx and trachea is the restoration of an adequate lumen of the respiratory tract with the formation of a laryngotracheal defect (LTD) and subsequent long-term stenting of the larynx and trachea. The final stage of surgical rehabilitation is a plastic closure of the LTD (3,4). 

The choice of method of plastic closure of LTD is primarily due to its size, the area of ​​respiratory lumen, as well as the state of the mucous membrane of the trachea and skin in the area of ​​the defect. 

Elimination of extensive defects of the larynx and trachea by using musculocutaneous plastics according to classical technique without the use of support materials is not always sufficient. 

In the latter case, in the postoperative period, conditions are often created for retraction and pathological flotation of the newly formed anterior tracheal wall and respiratory stenosis, which dictates the need to use frame tissues to close such defects (5-7). 

The implantation of various artificial materials does not lead to complete epithelialization of the larynx and trachea and subsequently, as a rule, is accompanied by the growth of granulations in the lumen of the latter, infection, inflammatory reaction of local tissues and rejection of implants (4). 

Costal cartilage is a promising material for autologous transplantation and is used to restore a defect in the cartilage of the nasal septum, auricle, tracheal rings, etc. the use of this autograft in modern reconstructive surgery of the neck organs. 

Costal cartilage plates with a thickness of 1 to 3 mm can be used in laryngotracheoplasty and plastic closure LTD.

## Case Report

Patient K., 31 years old, came to the clinic with diseases of the ear, throat and nose at Sechenov University with complaints about tracheostomy breathing. From the anamnesis, it is known that the patient underwent thyroidectomy regarding the nodular goiter of the thyroid gland. One month after surgical treatment, breathing difficulties began to be noted. Over the next 6 months, shortness of breath gradually increased with minimal physical exertion. She then went to the ENT clinic at the place of residence with these complaints, where she was diagnosed with bilateral paralysis and decompensated laryngeal stenosis during the examination. 

A lower tracheostomy was performed. During examination at the clinic of Sechenov University - breathing through the tracheostomy tube is free, the tracheostomy is under the arch of the cricoid cartilage.

On examination. 

According to laryngostroboscopy: the larynx mucosa is pink, the epiglottis is deployed in the shape of a petal, the pyriform sinuses are free, the vocal folds are in the middle position, while breathing and phonation are stationary, the width of the glottis on inspiration is 1 mm. The subglottic space is not visible. 

In our clinic we performed the extra-laryngeal lateral fixation of the right vocal fold with arytenoidotomy and resection of the cricoid cartilage arch (8). The period of stenting with a silicone T-tube in the postoperative period was 8 months.

After 8 months, the patient was re-hospitalized in our clinic for plastic closure of the larynx and trachea defect. Diagnosis: Chronic paralytic stenosis of the larynx. Condition after a strumectomy, tracheostomy, extra-laryngeal lateral fixation of the vocal fold with arytenoidotomy, resection of the cricoid cartilage arc, and T-tube endoprosthesis replacement. Laryngotracheal defect (LTD). 

**Fig 1:**
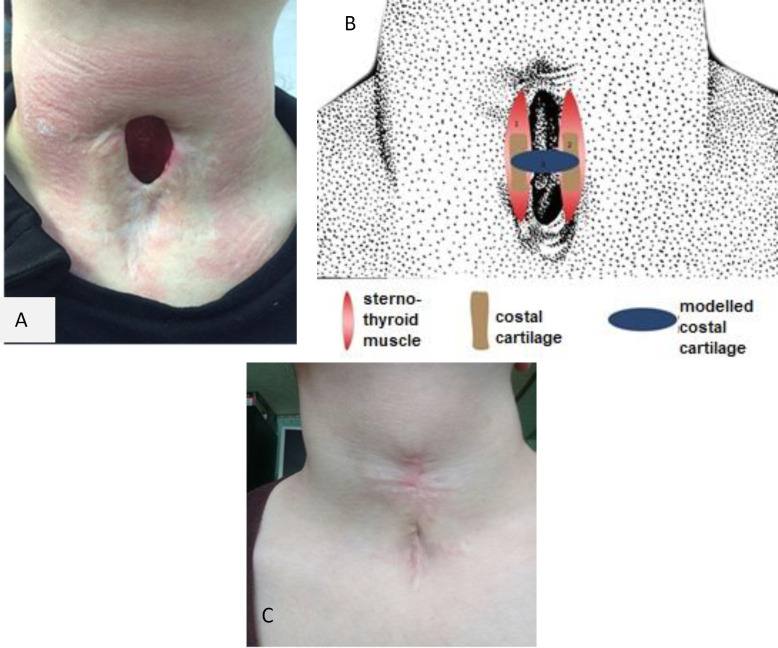
(a) Laryngotracheal defect of patient K.; (b) scheme of laryngotracheal defect plastics with a modeled costal auto-cartilage; (c) the patient’s K. neck 6 months after surgery

At the current admission patient complained about a defect on the front of the neck, hoarseness. Objectively: the patient's condition is satisfactory. The shape of the neck is not changed. On the front surface of the neck there is an LTD measuring 3.5 x 1.8 cm. The skin around the defect is scarred. (fig. 1a). With fibrolaryngoscopy: the vestibule of the larynx is not changed, the mucous membrane of the larynx and trachea is pink; the vocal folds are motionless –the right one in lateral position, left in the middle position. Rima glottidis - 7 mm, subglottic space is free. According to spirometry data, the ventilation ability of the lungs was not impaired before and after the operation.

For the first time in world clinical practice, plastic closure of an LTD with a ribbed auto-cartilage modeled by an erbium fiber laser was performed (9).

Surgery progress: the first step was the collection of costal cartilage from the region of the costal arch. 3 cartilaginous strips up to 0.5 cm thick, 3 cm long, and 1.5 cm wide were selected with a scalpel from the cartilaginous part of the 8th rib. One of the obtained cartilaginous fragments was modeled using an Erbium fiber laser wavelength of 1.56 μm (radiation is delivered by optic-thermo-mechanical contactor, the irradiation time of each point is 6 seconds, power 2.2 W) until a stable semi-oval shape is obtained.

 The second step was a bordering skin incision, 1 cm from the edge of the LTD. The separated skin flap was placed to form the front wall of the trachea and sutured along the midline with five Z-shaped sutures with Vicryl 3.0 threads. Muscle beds were formed from the sternum-thyroid muscles. 2 non-irradiated cartilage fragments 2.5 cm long and 0.5 cm thick were placed in the muscle beds and stitched along the distal edge with muscle PDS 2–0 filaments. 

A modeled by laser cartilage fragment was sutured on both sides to the medial edges of unirradiated cartilage fragments with PDS 2–0 filaments (fig. 1b). The skin around the defect was separated and the third layer was aligned in the midline above the formed muscle layer. Vycril 3.0 threads were used for muscle and skin sutures. 

After extubation, breathing through the natural airways was free. In the postoperative period, antibacterial and decongestant therapy was performed. The postoperative period was uneventful, skin sutures were removed on the 10th day. 

6 months after surgery, the patient noted the restoration of adequate breathing through the natural airways. The following studies were conducted to assess the sealing of the lumen of the larynx and trachea, functional viability, and to evaluate the preservation of the shape of auto-cartilage: MSCT and MRI of the neck organs, video fibrolaryngoscopy and laryngostroboscopy. Endoscopic picture of the larynx: the mucous membrane of the larynx is pink, vocal folds: the right one is in the lateral position, the left one is in the middle glottis is the width of the glottisis 7–8 mm. The flotation of the newly formed anterior tracheal wall was not determined (fig. 1 c).

**Fig 2:**
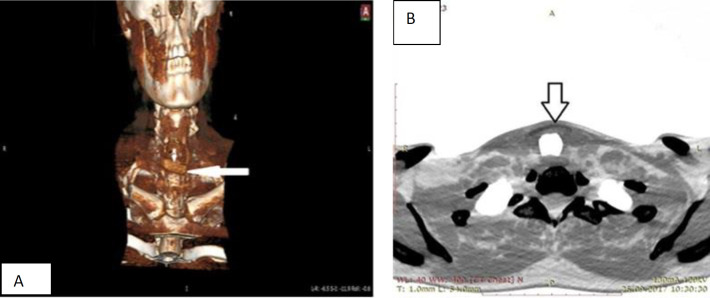
CT scan of neck organs in 2 frontal (a) and axial (b) projections 6 months after plastic closure LTD: cartilaginous autograft is indicated by an arrow

With a multi-layer spiral CT of the organs of the neck 6 months after surgery: condition after surgical interventions on the larynx, visualization of the cartilaginous autograft is noted. The semi-oval form of the autograft did not change; the cartilage tissue did not lysate with time (fig. 2a, b).

Twelve months after surgery under dynamic observation: there was no breathing impairment.

## Discussion

The main goal of open reconstructive surgery on the larynx and trachea is to restore the lumen of the respiratory tract through the formation of a laryngotracheal defect and subsequent stenting of the lumen of the larynx and trachea (10). Elimination of extensive defects of the larynx and trachea by using musculoskeletal plastics without the use of supporting materials is not always sufficient. Complications arise in the form of retraction and pathological flotation of the newly formed front wall of the neck and stenosis of the respiratory tract during breathing (11,12). The implantation of artificial materials does not lead to epithelization of the larynx and trachea and ends with an increase in granulation in the lumen of the hollow organs of the neck, accompanied by infection and rejection of implants (12–14).

Having studied the literature data, we concluded that for plastics of resistant LTD, it is preferable to use the body's own tissues. Depending on the size of the LTD, preference is given to costal auto-cartilage, ear cartilage and nasal septum cartilage, which, unlike autoconservatives, are better established, less often lysed and allow formation a stable hermetic lumen of the neck organs (12–15). 

The rib cartilage plates with a thickness of 1 to 3 mm can be used in laryngotracheoplasty and plastic closure of LTD. The strength of the costal cartilage, the ease of its extraction with minimal invasiveness and a sufficiently large volume of donor material make it possible receive it (13,16). However, the literature describes cases of excessive lysis and resorption and the inability to take the form of hollow organs of the neck (12,13).

In this regard, we first conducted an experimental study for the first time on 10 laboratory chinchilla rabbits. The goal was to develop and put into practice a method of plastic surgery using a rib auto-cartilage modeled by an erbium laser. In the experiment, the selection of optimal safe modes of exposure to cartilage tissue was carried out. The laser parameters used in the laser septochondrocorrection operation were taken as the basis (16,17). These modes allow you to give the necessary shape to the cartilage without violating its structure and biological properties.

## Conclusion

The method we demonstrated allows us to eliminate extensive defects of the larynx and trachea in one step by modeling the costal auto-cartilage with an erbium laser (λ = 1.56 μm), which is fixed to two non-irradiated costal fragments implanted in the sternum. The technique allows for providing the autograft with the necessary shape of a half ring and to avoid its resorption, as well as reduces the risk of postoperative complications.
